# Simultaneous quantification of 12 different nucleotides and nucleosides released from renal epithelium and in human urine samples using ion-pair reversed-phase HPLC

**DOI:** 10.1007/s11302-012-9321-8

**Published:** 2012-06-16

**Authors:** Alberto Contreras-Sanz, Toby S. Scott-Ward, Hardyal S. Gill, Jennifer C. Jacoby, Rebecca E. Birch, James Malone-Lee, Kevin M. G. Taylor, Claire M. Peppiatt-Wildman, Scott S. P. Wildman

**Affiliations:** 1 Department of Pharmaceutics, UCL School of Pharmacy, London, WC1N 1AX UK; 2 Medway School of Pharmacy, The Universities of Kent and Greenwich at Medway, Chatham Maritime, Kent, ME4 4TB UK; 3 Faculty of Life Sciences, London Metropolitan University, London, N7 8DB UK; 4 Research Centre for Clinical Science and Technology, UCL, London, N19 5LW UK

**Keywords:** Nucleotide, Nucleoside, HPLC, Solid-phase extraction, Ion-pair agent, Urine samples

## Abstract

Nucleotides and nucleosides are not only involved in cellular metabolism but also act extracellularly via P1 and P2 receptors, to elicit a wide variety of physiological and pathophysiological responses through paracrine and autocrine signalling pathways. For the first time, we have used an ion-pair reversed-phase high-performance liquid chromatography ultraviolet (UV)-coupled method to rapidly and simultaneously quantify 12 different nucleotides and nucleosides (adenosine triphosphate, adenosine diphosphate, adenosine monophosphate, adenosine, uridine triphosphate, uridine diphosphate, uridine monophosphate, uridine, guanosine triphosphate, guanosine diphosphate, guanosine monophosphate, guanosine): (1) released from a mouse renal cell line (M1 cortical collecting duct) and (2) in human biological samples (i.e., urine). To facilitate analysis of urine samples, a solid-phase extraction step was incorporated (overall recovery rate ≥ 98 %). All samples were analyzed following injection (100 μl) into a Synergi Polar-RP 80 Å (250 × 4.6 mm) reversed-phase column with a particle size of 10 μm, protected with a guard column. A gradient elution profile was run with a mobile phase (phosphate buffer plus ion-pairing agent tetrabutylammonium hydrogen sulfate; pH 6) in 2–30 % acetonitrile (*v/v*) for 35 min (including equilibration time) at 1 ml min^−1^ flow rate. Eluted compounds were detected by UV absorbance at 254 nm and quantified using standard curves for nucleotide and nucleoside mixtures of known concentration. Following validation (specificity, linearity, limits of detection and quantitation, system precision, accuracy, and intermediate precision parameters), this protocol was successfully and reproducibly used to quantify picomolar to nanomolar concentrations of nucleosides and nucleotides in isotonic and hypotonic cell buffers that transiently bathed M1 cells, and urine samples from normal subjects and overactive bladder patients.

**Electronic supplementary material:**

The online version of this article (doi:10.1007/s11302-012-9321-8) contains supplementary material, which is available to authorized users.

## Introduction

Extracellular nucleotides and nucleosides are released by most mammalian cells following mechanical and chemical stimuli, and in response to pathophysiological states such as inflammation and hypoxia [1]. Although numerous release mechanisms have been proposed to date [2, 3], these have proved challenging to define due to technical problems associated with quantifying nucleotide and nucleoside release. Once released, these molecules act at plasma membrane-bound purinoceptors (P1R and P2R) to initiate a sequence of downstream processes within the cell. The P1R family comprises four receptor subtypes (A1, 2A, 2B, 3), whereas the P2R family is divided into seven P2X subunits (P2X1–7) that form homo- or heteromeric ion channels and eight P2Y subtypes (P2Y1, P2Y2, P2Y4, P2Y6, and P2Y11–14), all G protein-coupled receptors [4]. Both P1R and P2R receptors are involved in a wide variety of physiological and pathophysiological processes in multiple organ systems ranging from digestion to neurotransmission, smooth muscle contraction, immune response, inflammation, and pain sensation [5]. Moreover, extracellular nucleotide and nucleoside signalling plays a key regulatory role in the kidney, especially in renal collecting duct tubular transport, and in regulating the medullary microcirculation [6–8].

In the bladder, nucleotides and nucleosides play an important role in sensing bladder filling and the ensuing detrusor muscle contraction that determines bladder voiding [9]. Interestingly, altered nucleotide and nucleoside signalling is thought to underlie pathological conditions of the bladder, such as overactive bladder (OAB) [10–12], interstitial cystitis [13, 14], and benign prostatic hyperplasia [10]. To date, the majority of studies have focused on the actions of adenosine (Ado) and adenosine triphosphate (ATP) on P1Rs and P2Rs, respectively, with little attention to other extracellular nucleotides or nucleosides. This is most likely due to due to the lack of appropriate assays to assess their concentration and/or biological function. Given that, like ATP and Ado, many of these other molecules are similarly degraded by endogenous ectonucleotidases and also exert their effect following binding to P1Rs and P2Rs [15–17], it is also important to investigate their extracellular concentration in model cell culture systems and biological samples such as blood and/or urine.

ATP concentration has historically been measured using the established luciferin–luciferase luminescence assay technique [18], which is highly sensitive and appropriate for measuring localized ATP. However, alternative analytical methods exist, including chromatographic systems such as high-performance liquid chromatography (HPLC) attached to a UV detector (HPLC-UV) [19–21], or liquid chromatography coupled to mass spectrometers (LC-MS) [22–25]. While disparity continues between the concentration of ATP measured close to the cell surface and that in the bulk extracellular phase, nanomolar or higher concentrations of a range of extracellular nucleotides and nucleosides have now been measured in a variety of systems including cultured cells [22], isolated tissues [21], food substances [20, 23, 24], fungi [25], and biological samples [19]. Despite this and recent studies describing ATP as a useful biomarker for certain diseases [10–14], the importance of other nucleotides and nucleosides remains largely unexplored, as indeed does their precise role in the physiology or pathophysiology of other organs or systems. Simultaneous quantification of ATP, adenosine diphosphate (ADP), adenosine monophosphate (AMP), Ado, guanosine triphosphate (GTP), guanosine diphosphate (GDP), guanosine monophosphate (GMP), guanosine (Gua), uridine triphosphate (UTP), uridine diphosphate (UDP), uridine monophosphate (UMP), and uridine (Uri) cannot be performed using the luciferin–luciferase assay, and, despite their known biological function, quantification of extracellular nucleotide/nucleoside concentrations in cell culture buffers or urine samples using a HPLC-UV-coupled system has yet to be reported.

The aim of this investigation was to develop a simple, fast, reliable, and reproducible method for quantifying up to 12 different nucleotides and nucleosides in samples of varying complexity (i.e., cell buffers and human urine samples). Specifically, we sought to determine whether differences in nucleotide and nucleoside concentrations could be detected between: (1) an isotonic and hypotonic buffer solution that transiently bathed M1 cells and (2) urine samples from OAB patients and non-OAB control subjects. The ion-pair reversed-phase, UV-coupled HPLC method described here was validated and successfully applied to quantify extracellular nucleotide and nucleoside concentrations in the above samples. This technique has the potential to be used not only in studies of lower urinary tract function under normal and disease states but also to assess changes in nucleotide and nucleoside levels in other biological and non-biological samples.

## Experimental

### Chemicals and reagents

ATP, ADP, AMP, Ado, GTP, GDP, GMP, Gua, UTP, UDP, UMP, Uri, ethanolamine, phosphoric acid, tetrabutylammonium hydrogen sulfate (TBAHS), K_2_HPO_4_, HClO_4_, NaCl, KCl, CaCl_2_, MgCl_2_, NaHCO_3_, HEPES, orthophosphoric acid, glucose, fetal bovine serum (FBS), penicillin/streptomycin, and sodium selenite/insulin/transferrin/ethanolamine were all obtained from Sigma-Aldrich (Poole, UK). HPLC-grade acetonitrile (ACN) and methanol were obtained from Fisher Scientific (Loughborough, UK). Dulbecco’s modified Eagle’s cell culture media (DMEM) was purchased from Invitrogen (Paisley, UK). Creatinine was measured using a modified Jaffe method (R&D Systems, Abingdon, UK). Deionized water (dH_2_O) was produced in-house.

### Apparatus and chromatographic conditions

The HPLC setup was formed of an Agilent 1200 series autosampler (G1329A), column oven (G1316A), degasser (G1379A), quaternary pump (G1311A), and UV detector (G1314B). Instrumental control, data acquisition, and processing were all carried out using ChemStation software (Agilent Technologies, Wokingham, UK).

A Synergi Polar-RP 80 Å 250 × 4.6 mm column fitted with a Synergi Polar-RP 80 Å guard column (Phenomenex, Macclesfield, UK) was used for chromatographic separation. The column was kept at 40 °C, and the detection absorption wavelength set at 254 nm. Strata X solid-phase extraction (SPE) cartridges (30 mg ml^−1^) were purchased from Phenomenex (Macclesfield, UK).

The mobile phase was adapted from a previous study [21] and consisted of a gradient mix of phosphate buffer (39 mM K_2_HPO_4_, 26 mM KH_2_PO_4_, and 10 mM TBAHS; solution A) adjusted to pH 6.0 with orthophosphoric acid and ACN (solution B) at concentrations that varied from 2 % to 30 % over the duration of the HPLC run as follows: 0 min (98 % solution A, 2 % solution B), 10 min (92 % solution A, 8 % solution B), 20 min (70 % solution A, 30 % solution B), 20.5 min (98 % solution A, 2 % solution B), 35 min (98 % solution A, 2 % solution B). The flow rate was set at 1 ml min^−1^, and an injection volume of 100 μl was used for all analyzed samples. The column was conditioned for 15 min prior to running the first and subsequent samples, by applying phosphate buffer in 2 % ACN to the column. Samples were kept at room temperature in the autosampler for no longer than 12-h before injection. Analyte stability was tested and showed no significant degradation over 12-h (not shown). To elute all remaining compounds from the column and increase its lifetime, the column was washed at the end of each sequence for 20 min using a gradient of ACN (2–50 %) in phosphate buffer. All compounds were identified on the basis of their retention time (Table 1).

**Table 1:** Analytical data for nucleotide and nucleoside detection. LoD and LoQ are expressed in nanomolars (nM) and picomoles (pmol) per 100 μl of sample, in parenthesis. Data are the mean of five separate experiments (*n* = 5)

Analyte	Retention time (min)	Slope	Intercept	*r* ^2^	LoD	LoQ
ATP	21.8	1,219	0.89	0.9984	2.70 (0.27 pmol)	9.03 (0.90 pmol)
ADP	18.7	1,343	1.91	0.9996	2.67 (0.26 pmol)	8.91 (0.89 pmol)
AMP	12.3	1,978	1.52	0.9999	2.50 (0.24 pmol)	8.02 (0.80 pmol)
Ado	8.7	3,245	0.41	0.9962	0.89 (0.08 pmol)	2.97 (0.29 pmol)
GTP	20.5	1,340	1.40	0.9996	1.93 (0.19 pmol)	6.44 (0.64 pmol)
GDP	14.4	1,853	12.59	0.9971	1.44 (0.14 pmol)	4.83 (0.48 pmol)
GMP	7.7	2,124	12.08	0.9999	2.07 (0.20 pmol)	6.90 (0.69 pmol)
Gua	5.5	3,266	88.66	0.9993	1.73 (0.17 pmol)	5.76 (0.57 pmol)
UTP	20.8	687	0.88	0.9995	4.32 (0.43 pmol)	14.42 (1.44 pmol)
UDP	13.2	728	1.03	0.9998	4.56 (0.45 pmol)	15.22 (1.52 pmol)
UMP	6.7	1,544	11.10	0.9998	3.17 (0.31 pmol)	10.58 (1.05 pmol)
Uri	4.5	1,215	50.76	0.9880	4.23 (0.42 pmol)	14.13 (1.41 pmol)

### Preparation of standard and working solutions

All solutions were assayed in 2 ml amber glass HPLC vials (Chromacol, Welwyn Garden City, UK) with inserts if necessary. Buffers were made up daily and kept at 4 °C until required. Stock solutions containing all nucleotides and nucleosides were prepared in dH_2_O at a concentration of 50 μg ml^−1^, and appropriate serial dilutions were made, to the lowest limit of detection (LoD). Original stock solutions were stable for up to 3 months when stored in the dark at −20 °C. For each experiment, serial dilutions of all standard analytes were freshly prepared from frozen stock (50 μg ml^−1^). To obtain the standard curves necessary for determining unknown sample concentrations for all compounds, standards were separated in parallel with all experimental samples. To ensure accuracy and precision of the method was maintained, working standard solutions of all 12 nucleotides and nucleosides were used to “spike” cell/urine media samples. Duplicate injections were performed for all assays.

### Cell buffer and patient urine samples

Immortalized M1 cells were used from passage 25 to 30 and maintained in six-well plates in DMEM media supplemented with: FBS (5 %), penicillin (100 units ml^−1^), streptomycin (100 μg ml^−1^), insulin (6.7 ng ml^−1^), transferrin (5.5 μg ml^−1^), selenium (5.5 μg ml^−1^), and ethanolamine (2 μg ml^−1^), in a humidified incubator at 37 °C and 5 % CO_2_/95 % O_2_ atmosphere. Media was refreshed every 48 h, and cells were passaged when confluent (approximately every 4 days). Experiments were only performed using confluent cells. Cell media volume and buffer treatments were kept constant at 1 ml well^−1^. In order to normalize nucleotide release (nmol mg^−1^ protein), cells were lysed after experimentation and the protein levels quantified. For comparison purposes, cells were treated with either an isotonic physiological buffer (in mM: 132.5 NaCl, 3.5 KCl, 1 CaCl_2_, 1 MgCl_2_, NaHCO_3_, 10 HEPES, 5 glucose; pH 7.4) or a hypotonic buffer (in mM: 72.5 NaCl, 3.5 KCl, 1 mM CaCl_2_, 1 MgCl_2_, NaHCO_3_, 10 HEPES, 5 glucose; pH 7.4), to stimulate further release of nucleotides and/or nucleosides. These experiments were carried out by (1) removing the media, (2) adding either an isotonic or hypotonic buffer onto the wells, and (3) immediately removing and snap-freezing the buffer, to −80 °C until further analysis. All steps were carried out with care to avoid any mechanical disruption to cells.

With written consent, urine samples were obtained from human patients diagnosed with OAB attending the Whittington Hospital (UCL, London, UK) by trained physicians. Appropriate asymptomatic non-OAB controls were selected from university and hospital staff. Samples were immediately snap-frozen and kept at −80 °C until the time of processing. Immediately prior to HPLC analysis, samples were thawed, sterile-filtered (0.22 μm), and subjected to SPE as described below.

### SPE treatment of urine samples

To remove proteins and polar contaminants from urine samples, they were subjected to an optimised SPE procedure [19]. Polymeric reversed-phase Strata-X SPE cartridges (30 mg ml^−1^) and a 24-position vacuum manifold (Phenomenex, Macclesfield, UK) were utilized. Throughout, a low-pressure vacuum (2–3 mmHg for approximately 2 min) was used to collect all eluted fractions in separate HPLC vials. Each cartridge was initially pre-conditioned with 1 ml dH_2_O. The undiluted sample (1 ml) was loaded onto the pre-conditioned SPE cartridges, vacuum applied, and the resulting flow-through collected as fraction 1 (F1). A wash solution (1 ml) containing 25 mM ethanolamine (pH 5) was subsequently added and the resulting eluate collected as fraction 2 (F2). Finally, to elute remaining nucleotides and nucleosides, a solution (1 ml) of 30 % methanol in 25 mM ethanolamine (pH 5) was flushed through the cartridge to obtain fraction 3 (F3). Different percentages of different analytes were eluted in all fractions, and therefore, all fractions were subjected to full HPLC analysis. All fractions were injected separately, and the sum of the results for all of them was used to obtain the final total amount of nucleotides and nucleosides present in the original urine samples. The effect of SPE treatment on recovery rates was obtained by comparing the levels of nucleotide and nucleoside standards recovered in all three fractions, with and without SPE pre-treatment. Urinary creatinine was measured according to the manufacturer’s instructions using a modified Jaffe method.

### Inhibition of nucleotidases in cell buffers

Cell buffers were immediately treated with perchloric acid (HClO_4_) to inactivate/inhibit nucleotidase activity, as previously described [23, 26]. Briefly, HClO_4_ (120 μl, 0.4 M) was added to each cell buffer sample (800 μl). The sample was then neutralized with KOH (172 μl, 1 M) and the pH adjusted to 7.0. Samples were centrifuged at 13,000 rpm for 5 min to remove precipitates, and the supernatant was transferred into tubes and immediately snap-frozen until further analysis.

### Method validation

The method was validated for: specificity, linearity, LoD, lower limit of quantification (LoQ), system precision, accuracy, intermediate precision, and recovery rates after SPE.

The specificity was assessed by analyzing endogenous interferences within the samples and by running chromatograms with single analytes versus chromatograms containing all analytes of interest. The linearity was assessed by calibration of eight-point standard curves in dH_2_O for all compounds, from 50 μg ml^−1^ down to their LoQ.

The LoD was defined as three times the signal-to-noise ratio, whereas the LoQ was set at ten times the signal-to-noise ratio [27]. System precision was tested by injecting the same standard sample into the column six times. Six different sets of urine samples and isotonic cell buffers were used and spiked at two different concentrations (250 and 1,500 ng per 100 μl sample) to investigate the intermediate precision and accuracy. The accuracy was calculated by comparing the expected concentrations with the actual measured concentrations of the samples spiked with all 12 nucleotides and nucleosides. The intermediate precision was assessed by determining the relative standard deviation $\left[ {{\text{RSD}}\left( \% \right) = \left( {{\text{SD}}/{\text{mean}}\,{\text{found}}} \right) \times 100} \right]$ of the replicate analyses.

Stability studies for all analytes were performed under conditions comparable to those encountered during sample preparation and HPLC analysis, i.e., standard analyte mixes in dH_2_O were kept at room temperature in the autosampler. Analyses were repeated every 12-h for a period of up to 36-h.

### Statistical analysis

Unless otherwise stated, all values are expressed as mean ± SEM (standard error of the mean). All experiments were repeated at least three times, with each value representing the average of duplicate injections. For statistical analysis, unpaired *t* tests were used to assess for significant differences between experiments and appropriate controls. Results were analyzed with Graph Pad Prism 5 (La Jolla, California, USA), and values of *P* < 0.05 were considered statistically significant.

## Results and discussion

### HPLC separation

A Synergi polar-RP column was chosen to separate various nucleotides and nucleosides. Nucleosides consist of a nitrogenous base and sugar, while nucleotides consist of a nitrogenous base, sugar, and phosphate group. The phosphate group on the nucleotide makes the compound extremely polar. The nitrogenous bases in the compounds used here are all purines which are heterocyclic aromatic organic compounds consisting of a pyrimidine ring fused to an imidazole ring. The best column separation of nucleosides and nucleotides depends upon the column’s phase. A column that provides low hydrophobicity and a high polar and aromatic selectivity enhances separation.

**Fig. 1:**
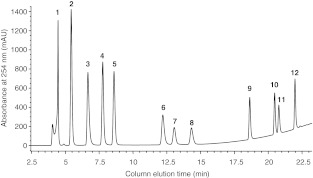
Representative chromatogram of a standard aqueous mixture of 12 nucleotides and nucleosides (50 μg ml^−1^) separated and analyzed using ion-pair reversed-phase HPLC coupled to a UV detector (set at 254 nm). Identities of the relevant peaks are: (*1*) Uri, (*2*) Gua, (*3*) UMP, (*4*) GMP, (*5*) Ado, (*6*) AMP, (*7*) UDP, (*8*) GDP, (*9*) ADP, (*10*) GTP, (*11*) UTP, (*12*) ATP

Two of the nucleosides analyzed, guanosine and adenosine, are highly polar in nature and are, in essence, not retained on reversed-phase columns by organic solvents. This problem can be overcome by running these compounds in very polar mobile phases, and, in this study, we used a buffer containing an ACN gradient (2–30 %). The mobile phase was also maintained at pH 6 to ensure all nucleotides and nucleosides remained in their uncharged forms, therefore increasing their retention time. However, at this pH, the lone pair of electrons on the nitrogen atoms may act as a base and interact with the silanol group in the stationary phase of the column to cause “peak tailing”. Hence, an ion-pairing agent (TBAHS) was added to the mobile phase in attempt to reduce this tailing for some of the nucleotides and nucleosides (Fig. 1). Agents such as TBAHS have been used in previous studies utilizing UV-coupled separation methods [19, 21].

Figure 1 shows an HPLC chromatogram for the separation of a standard aqueous mixture (50 μg ml^−1^) of all analytes of interest (ATP, ADP, AMP, Ado, GTP, GDP, GMP, Gua, UTP, UDP, UMP, and Uri). The HPLC method employed here provides a simple technique to simultaneously determine the concentration of all nucleotides and nucleosides using UV detection at 254 nm. We identified all analytes on the basis of their retention time (Table 1). Separation of other structurally related purine bases (e.g., uric acid, hypoxanthines, xanthines) was not attempted in this study. The total analysis time was 35 min per sample comprised of a column run time of 22 min and a post-run stabilization time of approximately 13 min. Although shorter stabilization periods have been reported in the literature [21], a time of less than 13 min resulted in substantial overlap of the GTP/UTP and UDP/GDP peaks in our hands. Despite this, 35 min is still substantially shorter than other published methods that analyzed multiple compounds in a single run (≥1 h) [20, 25, 28–30].

### Development of the urine sample SPE treatment

Biological samples often present with high levels of protein and polar contaminants, which makes the determination of nucleotide and nucleoside concentration difficult. A standard approach for eliminating proteins is to precipitate with organic solvents [31]. We attempted this by mixing equal volumes of ACN and sample and then filtering out the precipitates; this approach did not significantly improve the shape of the chromatogram, likely due to interfering polar compounds being retained in the samples. In other studies, reversed-phase SPE columns were used to pre-treat complex biological samples such as cerebrospinal fluid [19]. However, using Strata X SPE columns according to manufacturer’s recommendations, a high proportion of analytes were retained leading to low recovery values. Consequently, we optimized the protocol by pre-conditioned the columns by first adding dH_2_O, after which samples were loaded, and the flow-through collected as F1. Elution of nucleotides and nucleosides was evaluated by addition of 25 mM ethanolamine at three different pH values: 5.0, 6.0, and 7.0. The resulting elution fractions were collected as F2. All remaining compounds were then eluted using 30 % methanol in the different ethanolamine solutions (pH 5, 6, and 7), constituting F3. The three fractions were injected into the HPLC system separately and the results pooled to calculate the total amount of nucleotides and nucleosides present in the original samples.

As shown in Table 2, optimal results were obtained for both F2 and F3 when using ethanolamine at pH 5.0. Recovery values ranged from 98.8 % to 104.4 % with the exception of Uri which yielded 130.8 %. Unusually high yields may be explained by the presence of other low-molecular-weight compounds remaining in the samples and forming complexes with the nucleotides and nucleosides to give a higher UV-absorbance than expected [19]. The recovery values for the other nucleotides and nucleosides present suggest it is unlikely that Uri is co-eluting or co-determining with other nucleotide/nucleoside analytes. However, the small peak observed immediately ahead of the Uri peak (see Fig. 1) is potentially contributing to the unusually high yield obtained for this analyte. Hence, to control for any inaccuracies, all final SPE yields were normalized to correct for these values. This peak was not further investigated. F1 and F2 contained different proportions of nucleotides and nucleosides, whereas F3 primarily contained Ado (results not shown), and therefore all three individually analyzed fractions were pooled into one final value.

**Table 2:** Total recovery of nucleotides and nucleosides from urine samples spiked with a standard mixture of all compounds of interest (1,500 ng) after Strata X SPE pre-treatment

Analyte	Recovery (%) pH 5.0	Recovery (%) pH 6.0	Recovery (%) pH 7.0
ATP	100.2	100.9	99.6
ADP	99.9	104.0	100.2
AMP	98.8	100.8	99.1
Ado	99.4	102.2	99.9
GTP	100.5	105.6	106.3
GDP	104.4	108.5	111.2
GMP	99.2	101.4	98.8
Gua	100.4	102.7	102.4
UTP	103.8	106.0	105.5
UDP	100.8	103.1	102.4
UMP	99.0	100.9	80.7
Uri	130.8	132.4	131.9

Data shown summarizes the overall recovery achieved in all three pooled fractions (F1, 2, and 3). The recovery of analytes in F2 and F3 was tested using 25 mM ethanolamine at three different pH values (5.0, 6.0, 7.0) and the results pooled together with fraction 1. Data are the mean of three separate experiments (*n* = 3)

### Method validation

**Fig. 2:**
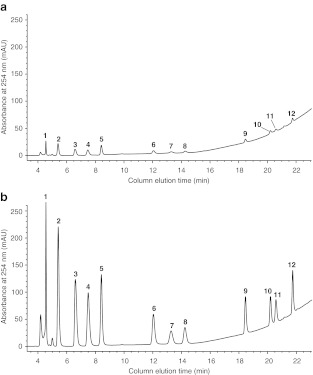
Representative HPLC-UV chromatograms of (**a**) normal hypotonic cell buffer sample and (**b**) hypotonic cell culture buffer sample spiked with a standard mixture of all analytes of interest (1,500 ng). Identities of the relevant peaks are: (*1*) Uri, (*2*) Gua, (*3*) UMP, (*4*) GMP, (*5*) Ado, (*6*) AMP, (*7*) UDP, (*8*) GDP, (*9*) ADP, (*10*) GTP, (*11*) UTP, (*12*) ATP

The specificity of the method was tested for endogenous interference from samples. Although urine samples presented interfering peaks and were first purified by SPE, cell buffers did not, and hence, were analyzed directly by HPLC-UV (as shown in Fig. 2).

Calibration curves were obtained by using linear regression analysis of eight-point concentration curves (not shown). The regression parameters, including slope, intercept, and correlation coefficient (*r*), are shown in Table 1. The *r* values were good (0.9999 for AMP to 0.9880 for Uri), and linearity was checked for the range of concentrations from 50 μg ml^−1^ down to the LoQ of the respective nucleotides and nucleosides. LoD values ranged from as low as 0.89 nM for Ado up to 4.56 nM of UDP (Table 1). LoQ values were found to be from 2.97 nM for Ado up to 15.22 nM for UDP (Table 1). LoQ values established here are substantially lower than those reported in previous studies using a coupled-column [32], or standard UV-coupled HPLC [28, 32, 33]. Specifically, the values for ATP, ADP, Ado, GTP, GDP, and GMP are one order of magnitude lower than those of one recent publication [21]. Moreover, although not all relevant nucleotides and nucleosides were analyzed, a recent LC-MS-based study by Buescher and colleagues reported similar LoQ values for ATP, ADP, AMP, GTP, and GMP to those found here [34]. Additional pre-treatment of samples (e.g., drying–resuspension or lyophilization) could be beneficial in cases in which the analytes of interest are below the LoQ of the technique presented here.

To check the system’s precision, six replicate analyses of the standards were carried out. The percentage RSD ranged between 0.08 and 0.87 indicating satisfactory precision for analysis [27].

The accuracy of the protocol was determined for each analyte by performing recovery analysis of six cell buffer samples independently spiked with two different concentrations of standards containing all 12 nucleotides and nucleosides (Fig. 2). As shown in Table 3, the accuracy varied from 80.66 % up to 115.20 %, a satisfactory level for this method. Moreover, the intermediate precision of the method (1.22 % and 11.48 % RSD) was also found to be satisfactory [27].

**Table 3:** Accuracy and intermediate precision of the HPLC-UV separation method

Compound	Spiked (250 ng)		Spiked (1500 ng)	
	Accuracy (%)	RSD (%)	Accuracy (%)	RSD (%)
ATP	93.32	3.15	93.32	2.23
ADP	92.39	10.67	93.42	5.33
AMP	92.87	4.37	93.31	1.30
Ado	97.35	2.47	93.34	1.93
GTP	97.10	11.48	87.33	9.89
GDP	102.58	3.22	96.52	2.70
GMP	100.48	1.77	95.32	1.89
Gua	115.20	5.60	85.24	2.82
UTP	92.24	11.48	95.84	5.75
UDP	112.37	1.22	99.43	2.76
UMP	100.76	1.67	95.96	1.84
Uri	80.66	3.10	82.96	5.91

Isotonic cell buffer samples were spiked with either 250 or 1,500 ng of a standard mixture of all nucleotides and nucleosides of interest. For further information, see the “Results and discussion.” Data are the mean of six separate experiments (*n* = 6)

In order to simulate the sample preparation and autosampler conditions, the stability of all nucleotides and nucleosides was assessed by storing standard mixtures at room temperature and then analyzing them at 12-h intervals over a 36-h period. All compounds remained stable for up to 12-h in these conditions, whereas some nucleotides (i.e., ATP, AMP, and GTP) showed significant degradation or higher levels (i.e., UTP, UDP, GMP, and Gua) after 12- and 24-h intervals (not shown). This indicates that samples should not be left at room temperature, or in the autosampler, for longer than 12-h prior to analysis. If this cannot be achieved in future studies, it is recommended that a refrigerated autosampler be employed.

### Inhibition of nucleotidases

Some biological fluids and samples may contain membrane-bound and/or soluble nucleotidases [35]. To prevent enzymatic degradation of nucleotides and nucleosides, nucleotidase activity was inhibited by acid-treatment [23, 26]. To restore a neutral pH to samples, the HClO_4_ was neutralized with KOH, producing a precipitate that needed to be removed from the samples by centrifugation. It is likely that, despite our efforts to completely remove these precipitates, they continued to form over time as compounds adsorbed to the remaining perchlorate [36]. It was noted that, for some analytes, this treatment increased the area under the peak and modified the retention times, and hence, we normalized for these effects by also treating the standards with HClO_4_/KOH. Alternative methods for inhibiting nucleotidases are available. For example, focused microwave radiation has been shown to rapidly and irreversibly inactivate nucleotidases [37] and several ectonucleotidase inhibitors, including ARL67156 are available [38]. Unfortunately, the relatively high cost of such inhibitors precludes their use in large-scale studies. Heat inactivation has been used to treat tissue culture samples, as adenosine nucleotides can withstand the high temperatures required to inactivate nucleotidases [39].

### Analysis of in vitro samples: nucleotide and nucleoside release from a renal cell line

Epithelial cells are known to release adenosine and uridine nucleotides under both basal and stimulated conditions. Tatur and colleagues used an ion-pair reversed-phased HPLC technique to quantify extracellular nucleotides and nucleosides released from A549 lung epithelial cells [40]. They showed ATP, ADP, UTP, and UDP release were all increased following hypotonic shock, with no changes in AMP and Ado levels. Primary cultures of human renal epithelia (proximal tubule, mixed cortical and polycystic kidney cells) have also been shown to release ATP and other nucleotides, including diadenosine pentaphosphate and hexaphosphate after hypotonic stimulation [41, 42]. Moreover, Schwiebert et al. reported the release of low concentrations (nanomolars to picomolars) of ATP from native cortical collecting duct (CCD) cells that increased after hypotonic stimulation [43]. Once normalized to cellular protein levels (a measure of cell number), our results indicated that all 12 nucleotides and nucleosides were present in isotonic and hypotonic buffers that had been in contact with the cells (Table 4). To ensure that the differences in levels of nucleotides and nucleosides following stimulation were unlikely to originate from degradation pathways, but from immediate release from cells, nucleotidases were inhibited by acid-treating samples immediately prior to analysis. Cell viability was confirmed by trypan blue exclusion staining and morphological assessment (data not shown).

**Table 4:** Nucleotide and nucleoside release from M1 renal cells under isotonic and hypotonic buffer conditions

Compound	M1 CCD isotonic buffer	M1 CCD hypotonic buffer
ATP	0.39 ± 0.10	1.20 ± 0.25^a^
ADP	0.11 ± 0.03	0.20 ± 0.03
AMP	0.08 ± 0.01	0.34 ± 0.08^a^
Ado	0.08 ± 0.01	0.30 ± 0.10^a^
GTP	0.51 ± 0.08	0.35 ± 0.01
GDP	0.19 ± 0.02	0.087 ± 0.004^a^
GMP	0.10 ± 0.06	0.40 ± 0.05^a^
Gua	0.11 ± 0.02	0.67 ± 0.18^a^
UTP	0.63 ± 0.10	0.86 ± 0.39
UDP	0.07 ± 0.03	0.21 ± 0.08
UMP	0.21 ± 0.04	0.60 ± 0.17^a^
Uri	0.74 ± 0.12	0.88 ± 0.26

Data are expressed as nM µg^−1^ protein and are means ± SEM (*n* = 18)

^a^Values that are significantly different from isotonic buffer (unpaired *t* test; *P* < 0.05). For further information, see the “Results and discussion”

To the best of our knowledge, our study is the first to simultaneously measure an increase in ATP, AMP, Ado, GMP, Gua, and UMP and a decrease in GDP, following hypotonic challenge of a renal cell line. No changes were observed in ADP, GTP, UTP, UDP, and Uri levels (Table 4). In general agreement with previous observations of low nanomolar release in basal conditions [43], the ATP concentration, prior to normalization to the protein level, ranged from 110 nM under basal (isotonic) conditions to 370 nM after hypotonic stimulation. However, these values are lower than those reported in some studies (0.5–2 μM; [42]). This discrepancy may be explained by differences in cell type used, cell numbers, or the degree of increase in hypotonicity. All publications concur in reporting higher levels of ATP release following hypotonic stimulation, a condition likened to the process of stretch in the lumen of the nephron.

Due to the complexity and wide distribution of P1Rs and P2Rs in the kidney, the physiological significance of increased levels of nucleotides and nucleosides remains largely undefined. A recent review highlighted the implications for regulation of transport mechanisms for sodium, potassium, and other ions [44]. Others have proposed that the renal hemodynamic function and microvasculature are also regulated by P1Rs and P2Rs [6, 7]. This HPLC technique will facilitate further in vitro and in vivo studies into the role of multiple nucleotides and nucleosides in kidney function.

### Analysis of biological samples: urine from OAB patients

Historically, nucleotide analysis of the urine of cancer patients has shown differences in levels of some purines and pyrimidines in comparison to those of healthy controls [30, 45–47]. These compounds may originate from either increased turnover of different types of RNA [47], or from extracellular release from the urinary tract [48–51]. Altered nucleotide and/or nucleoside signalling is also implicated in several other bladder diseases, including OAB [10–14]. In this study, we use our optimized HPLC-UV method to investigate: (1) the viability of detecting all 12 compounds of interest in the urine of OAB and asymptomatic non-OAB control patients and (2) whether urinary ATP and other nucleotides and/or nucleosides levels is altered in the cohort of OAB patients. All analyte levels were normalized to urinary creatinine levels to account for differences in urine concentration [30]. As shown in Table 5, all 12 nucleotides and nucleosides were detected in the urine of OAB patients and controls. However, large variations in some of the analytes were observed between individuals, even within the same sample group. A similar level of variation has been reported in the levels of other urinary nucleotides and nucleosides from cancer patients [45]. Despite an apparent trend towards higher levels of urinary AMP, GTP, GMP, UDP, and Uri in OAB patients, the only significant changes in the urine of these patients were an increase in Ado, and a decrease in UMP (*P* < 0.05; *n* ≥ 11). The variation within patient groups is most likely affecting the significance levels, and thus larger groups will be required to increase the statistical power of further studies.

**Table 5:** Levels of nucleotides and nucleosides in urine from OAB patients and asymptomatic non-OAB controls

Compound	Asymptomatic non-OAB	OAB
ATP	19 ± 12	38 ± 18
ADP	194 ± 73	165 ± 26
AMP	31 ± 5	128 ± 47
Ado	71 ± 48	2,840 ± 1094^a^
GTP	500 ± 146.5	1,429 ± 630
GDP	1,157 ± 577	1,312 ± 534
GMP	55 ± 18	283 ± 110
Gua	4,890 ± 2039	3,690 ± 1149
UTP	225 ± 144	190 ± 69
UDP	241 ± 80	464 ± 259
UMP	2,260 ± 816	908 ± 260^a^
Uri	192 ± 92	486 ± 155

Data are expressed as pM mg^−1^ creatinine dL^−1^ and are means ± SEM (*n* = 33 for OAB; *n* = 11 for non-OAB controls)

^a^Values that are significantly different from asymptomatic non-OAB control (unpaired *t* test; *P* < 0.05). For further information, see the “Results and discussion”

P1Rs are widely expressed throughout all layers of the urothelium, and while their role remains largely unexplored, a previous study suggested P1Rs modulate exocytosis in umbrella cells [52]. We hypothesize that increased urinary levels of Ado in the OAB group could trigger exocytotic mechanisms, which in turn may cause the exacerbated release of ATP from bladder urothelial cells. This increased urothelial ATP release would ultimately activate P2Rs in sensory afferents, leading to hyper-stimulation and the symptoms of OAB [53]. Understanding the effect of decreased UMP and other extracellular guanosine and uridine nucleotides in the urinary tract will require further investigation.

### Practical application

The method described in this study is rapid and direct, and readily performed using standard HPLC apparatus. Sample preparation and HPLC fractionation and analysis can all be performed in 12-h or less at room temperature without concern for nucleotide/nucleoside degradation, and hence, this method has the potential to be applied in wide variety of research and clinical environments. Where ready access to HPLC is not available, this technique offers a clear opportunity for cost-effective (<£5 per sample; see Online resource 1 in Electronic supplementary material) and focused collaborative research between clinical facilities and more chemically/pharmacologically oriented laboratories, such as our own.

## Conclusion

In this study, we describe a HPLC-UV method that permits simultaneous quantification of 12 different nucleotides and nucleosides in in vitro and biological samples. Validation of the method indicates it has good specificity, linearity, LoQ, LoD, system precision, accuracy, and intermediate precision. The sensitivity of the method is similar to, or better than, previous reports, yet the number of analytes per run are significantly higher than similar systems using the same detector and provides shorter run times. The protocol was successfully used to detect differences in low levels of nucleotides and nucleosides in all samples. Moreover, this method can be further adapted to detect other relevant endogenous nucleotides and nucleosides of interest. This analytical technique may not only contribute to our understanding of the physiology and pathophysiology of the lower urinary tract but also prove particularly useful in studying nucleotide and nucleoside levels in other organs or systems.

## Electronic supplementary material

Below is the link to the electronic supplementary material.

ESM 1 (PDF 30.6 kb)
