# Supplementary material for: Simultaneous quantification of 12 different nucleotides and nucleosides released from renal epithelium and in human urine samples using ion-pair reversed-phase HPLC
Source: Purinergic Signal. 2012 Jun 16;8(4):741–51. doi: 10.1007/s11302-012-9321-8 (PMC3486167; doi:10.1007/s11302-012-9321-8)
Supplement: Supplementary file 1 — (PDF 30.6 kb) [file 11302_2012_9321_MOESM1_ESM.pdf]

Online Resource 1

Simultaneous quantification of 12 different nucleotides and nucleosides released from renal epithelium and in human urine samples using ion-pair reversed-phase HPLC

Journal: **Purinergic Signalling**

Alberto Contreras-Sanz[1], Toby S. Scott-Ward[2], Hardyal S. Gill[1], Jennifer C. Jacoby[3], Rebecca E. Birch[2], James Malone-Lee[4], Kevin M.G. Taylor[1], Claire M. Peppiatt-Wildman[2], Scott S. P. Wildman[2]

[1]Department of Pharmaceutics, UCL School of Pharmacy, London; [2]Medway School of Pharmacy, The Universities of Kent and Greenwich at Medway; [3]Faculty of Life Sciences, London Metropolitan University, London; [4]Research Centre for Clinical Science and Technology, UCL, London; UK

Corresponding authors: **Toby S. Scott-Ward** or **Scott S. P. Wildman**

Medway School of Pharmacy, The Universities of Kent and Greenwich at Medway, Chatham Maritime, Kent, ME4 4TB, UK.

E-mail: t.s.scott-ward@kent.ac.uk or s.s.wildman@kent.ac.uk

Online Resource 1

**Item**	**Cost (£)**	**Samples analysed**	**Cost per sample (£)**
Column[*]	700	500	1.4
Reagents[**]	500	250	2
Consumables[***]	400	400	1
**Total**	**1600**	**-----**	**4.4**

Supplementary Table 1. Basic research costs anticipated for analysis of human urine and related biological samples for nucleotides/nucleoside levels using the HPLC-UV method described in this study. Note that this costing *excludes* additional variables such as the operator/researcher, sample acquisition/preparation, adaption of the method and system maintenance and overhead costs (specific knowledge of the facilities available) and is likely to be an underestimate. Where ready access to HPLC is not available, this analysis cost per sample estimate can only be realised through appropriately-funded collaborative research between clinical/biological facilities and more chemically/pharmacologically-oriented laboratories. [*] Including Synergi 4 μm Polar-RP 80 Å column with column guard (Phenomenex Ltd, product no. 00G-4336-E0) and Strata-X RP SPE cartridges (product no. 8B-S100-TAK); [**] including nucleotide/nucleoside standards, organic solvents (ACN, methanol), buffers (HEPES, TBAHS) and other chemicals; [***] including plastic-ware (tips, tubes, etc), and other non-chemical consumables.
